# Supplementary material for: Sobrerol Improves Memory Impairment in the Scopolamine-Induced Amnesia Mouse Model
Source: Int J Mol Sci. 2025 May 12;26(10):4613. doi: 10.3390/ijms26104613 (PMC12111148; doi:10.3390/ijms26104613)
Supplement: Supplementary file 1 [file ijms-26-04613-s001.zip › ijms-3597697-SI.pdf]

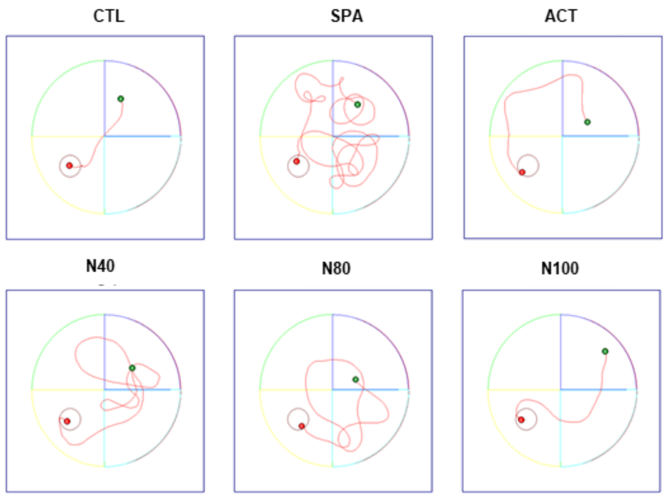

Figure S1. Morris Water Maze Test representative track map (Day 27). CTL (saline, vehicle control); SPA (2 mg/kg/day scopolamine, negative control); ACT (2 mg/kg/day Aricept, positive control); N40 (40 mg/kg/day NRM-331); N80 (80 mg/kg/day NRM-331); and N100 (100 mg/kg/day NRM-331). Green spot: Starting point, Red spot: Ending point.

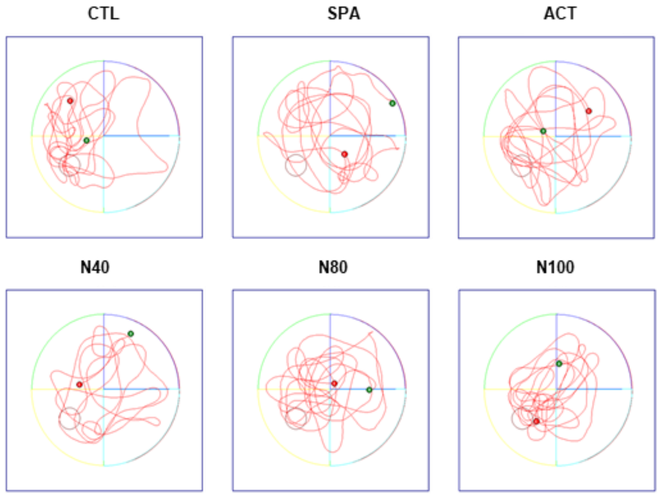

Figure S2. Morris Water Maze Test representative probe trial track map Day 28. CTL (saline, vehicle control); SPA (2 mg/kg/day scopolamine, negative control); ACT (2 mg/kg/day Aricept, positive control); N40 (40 mg/kg/day NRM-331); N80 (80 mg/kg/day NRM-331); and N100 (100 mg/kg/day NRM-331). Green spot: Starting point, Red spot: Ending point.
